# Alterations in left ventricular function during intermittent hypoxia: Possible involvement of O-GlcNAc protein and MAPK signaling

**DOI:** 10.3892/ijmm.2015.2198

**Published:** 2015-04-24

**Authors:** XUELING GUO, JIN SHANG, YAN DENG, XIAO YUAN, DIE ZHU, HUIGUO LIU

**Affiliations:** Department of Respiratory and Critical Care Medicine, Tongji Hospital, Huazhong University of Science and Technology, Key Laboratory of Respiratory Disease of the Ministry of Health, Wuhan 430030, P.R. China

**Keywords:** intermittent hypoxia, O-GlcNAcylation, left ventricular function, p38 mitogen-activated protein kinase, extracellular signal-regulated kinase 1/2

## Abstract

Obstructive sleep apnea, characterized by recurrent episodes of hypoxia [intermittent hypoxia (IH)], has been identified as a risk factor for cardiovascular diseases. The O-linked β-N-acetylglucosamine (O-GlcNAc) modification (O-GlcNAcylation) of proteins has important regulatory implications on the pathophysiology of cardiovascular disorders. In this study, we examined the role of O-GlcNAcylation in cardiac architecture and left ventricular function following IH. Rats were randomly assigned to a normoxia and IH group (2 min 21% O_2_; 2 min 6–8% O_2_). Left ventricular function, myocardial morphology and the levels of signaling molecules were then measured. IH induced a significant increase in blood pressure, associated with a gradually abnormal myocardial architecture. The rats exposed to 2 or 3 weeks of IH presented with augmented left ventricular systolic and diastolic function, which declined at week 4. Consistently, the O-GlcNAc protein and O-GlcNAcase (OGA) levels in the left ventricular tissues steadily increased following IH, reaching peak levels at week 3. The O-GlcNAc transferase (OGT), extracellular signal-regulated kinase 1/2 (ERK1/2) and the p38 mitogen-activated protein kinase (p38 MAPK) phosphorylation levels were affected in an opposite manner. The phosphorylation of calcium/calmodulin-dependent protein kinase II (CaMKII) remained unaltered. In parallel, compared with exposure to normoxia, 4 weeks of IH augmented the O-GlcNAc protein, OGT, phosphorylated ERK1/2 and p38 MAPK levels, accompanied by a decrease in OGA levels and an increase in the levels of myocardial nuclear factor-κB (NF-κB), inflammatory cytokines, caspase-3 and cardiomyocyte apoptosis. Taken together, our suggest a possible involvement of O-GlcNAc protein and MAPK signaling in the alterations of left ventricular function and cardiac injury following IH.

## Introduction

The O-linked β-N-acetylglucosamine (O-GlcNAc) modification (O-GlcNAcylation) of cytoplasmic and nuclear proteins has been shown to have important regulatory implications on the pathophysiology of cardiovascular disorders (1,2). O-GlcNAcylation is catalyzed by the enzyme, O-GlcNAc transferase (OGT), which is responsible for the addition of O-GlcNAc using substrate UDP-N-acetylglucosamine (UDP-GlcNAc), which is reversed by the complementary O-GlcNAcase (OGA) for the hydrolytic cleavage of O-GlcNAc (3). O-GlcNAcylation modifies the same or neighbouring serine/threonine sites as phosphorylation. A complex and extensive cross-talk exists between O-GlcNAcylation and phosphorylation. OGT and OGA form transient complexes with kinases and phosphatases (4). Moreover, globally increased O-GlcNAcylation is known to influence the phosphorylation dynamics in a site-specific manner, resulting in either a reduction or augmentation of phosphorylation (5).

The post-translational modification by O-GlcNAcylation acts as a critical regulatory mechanism for numerous biological processes involving a range of target proteins, including kinases, phosphatases, transcriptional factors and signal transduction molecules (1,4,6). Studies have demonstrated that the acute augmentation of O-GlcNAc levels improves the tolerance to stress stimuli (7), confers cardioprotective effects against ischemia-reperfusion (IR) injury (8), blunts the cardiomyocyte nuclear factor of activated T-cells (NFAT)-induced changes during hypertrophy (9) and attenuates vascular inflammation (10). Conversely, a sustained increase in O-GlcNAcylation contributes to the development of vascular and cardiac dysfunction (1,2). Its ability to regulate reactive oxygen species (ROS) generation, calcium homeostasis (7,11,12), nuclear factor-κB (NF-κB) activation, the production of inflammatory cytokines (13,14) and intracellular signaling pathways, such as mitogen-activated protein kinases (MAPKs) and NFAT (1,9) may underlie the protective or negative effects of O-GlcNAcylation on the cardiovascular system.

Obstructive sleep apnea (OSA) has been identified as a risk factor for cardiovascular diseases, including myocardial infarction, hypertension and stroke, resulting from oxidative stress and augmented inflammatory responses caused by a recurrent decrease in arterial oxygen [intermittent hypoxia (IH)] (15). Animal subjected to long-term IH have been shown to exhibit myocardial apoptosis, cardiac hypertrophy, pathological remodeling and an impairment in global cardiac function (16,17). Post-translational modifications, particularly phosphorylation, are important in mediating the OSA-associated pathological manifestations by functionally regulating a subset of proteins involved in signaling pathways, transcriptional factor activation, neurotransmitter synthesis and cardioprotection in rodents (18). Members of the MAPK family, primarily extracellular signal-regulated kinase 1/2 (ERK1/2) and p38 MAPK participate in cardiovascular pathophysiology processes related to OSA by regulating the systemic inflammation and redox status (16,19). Given the resemblance between the periodic reoxygenation in IH and IR injury, during which the processes of the overproduction of ROS and the enhancement of inflammatory reactions contribute to organ damage, in study, we aimed to determine whether protein O-GlcNAc levels are related to the impairment in left ventricular (LV) function and the associated pathological hypertrophy in the heart in a rat model prone to the development of hypertension (20).

## Materials and methods

### IH procedures

Male Wistar rats (weighing 200–220 g, 8 weeks old) were provided by the Experimental Animal Center of Tongji Hospital, Huazhong University of Science and Technology, Wuhan, China. The rats were housed under specific pathogen-free (SPF) conditions on a 12-h light-dark cycle with free access to regular chow and water. All protocols were approved by the Animal Care and Use Committee of Huazhong University of Science and Technology.

The normoxia and IH protocols were performed as described in our previous study with some modifications (20). The rats were randomly assigned and exposed to either normoxia (21% O_2_) or IH (alternating from 2 min 21% O_2_ to 2 min 6–8% O_2_) from 8:00 a.m. to 4:00 p.m. for 4 weeks [4 weeks normoxia (CON); 4 weeks IH (CIH)]. The desired gas profile was achieved by a computerized system (BioSpherix OxyCycler A84; BioSpherix Ltd., Lacona, NY, USA) to control the distribution of pure nitrogen or oxygen into the chambers. Weekly, the rats were weighed, and several rats from each group were anesthetized with urethane [1.2 g/kg, by intraperitoneal (i.p.) injection] for arterial blood pressure measurements and cardiac catheterization. In parallel, rats were anesthetized and 3 arterial blood samples were drawn from the right carotid artery prior to exposure to IH (baseline, C0), at the most hypoxic portion of the cycle (I2) and at the peak of the reoxygeneration (C2), respectively. Arterial blood gas assessments were performed using an ABL5 blood gas analyzer (Radiometer, Copenhagen, Denmark).

### Cardiac catheterization and remodeling index

Cardiac hemodynamics measurements were conducted according to previous studies (20,21). Briefly, the rats were anesthetized with spontaneous respiration. A polyethylene cannula (PE-50) was introduced into the right carotid artery and advanced into the left ventricle. The heart rate (HR), LV end-systolic pressure (LVESP) and LV end-diastolic pressure (LVEDP) were monitored using a PowerLab/4SP data acquisition system (ML118; AD Instruments, Medford, MA, USA). The maximal rates of LV systolic and diastolic pressure (±dp/dt) were calculated. Thereafter, the rats were sacrificed by exsanguination. The hearts were removed and placed in ice-cold phosphate-buffered saline (PBS) solution, blotted and weighed. The heart weight-to-body weight index (HW/BW) and the left ventricule plus septum weight-to-body weight index (LVW/BW) were calculated to measure the degree of LV hypertrophy. The LV lateral-mid free wall was fixed in 4% formaldehyde and embedded in paraffin. A portion of the left ventricle was immediately frozen in liquid nitrogen and stored at −80°C.

### Histological analysis

LV free wall sections (5-*µ*m-thick) were stained with hematoxylin and eosin (H&E) or Masson’s trichrome. The cardiomyocyte diameter and myocardial interstitial collagen content were quantitatively analyzed using Image-Pro Plus 7.0 software (IPP; Media Cybernetics, Silver Spring, MD, USA) as previously described (22,23). Briefly, for the cardiomyocyte diameter assessment, 100 cells with a round shape and central nuclei randomly selected from 5 regions of each slide (×200 magnification) were analyzed. The total myocardial interstitial collagen area, excluding the perivascular collagen was quantitatively determined in Masson’s trichromestained sections (×200 magnification).

### Terminal deoxynucleotidyl transferase-mediated dUTP nick-end labeling (TUNEL) assay

Cardiac apoptosis was determined by TUNEL assay using the *In Situ* Cell Death Detection kit (Roche, Mannheim, Germany) following the instructions provided by the manufacturer. Briefly, following deparaffinization and rehydration, the sections were treated with proteinase K (20 *µ*g/ml) for 15 min. The slides were then immersed in TUNEL reaction mixture at 37°C for 1 h in a humidified atmosphere in the dark. A total of 10 random fields per tissue section were captured at ×200 magnification (BX51 microscope; Olympus, Tokyo, Japan) and analyzed using IPP software. The apoptotic index (%) was calculated as the ratio of the number of TUNEL-positive nuclei divided by the total number of nuclei.

### Immunohistochemistry (IHC)

IHC was carried out on the paraffin-embedded LV sections (5-*µ*m-thick) using a 3,3′-diaminobenzidine (DAB) kit (Dako, Copenhagen, Denmark) following the manufacturer’s instructions. Briefly, the sections were deparaffinized, blocked with 3% hydrogen peroxide, treated with 3% bovine serum albumin (BSA) in PBS (pH 7.4), and incubated with the primary antibodies specific for caspase-3 (1:50), NF-κB p65 (1:100), OGA (1:100) or OGT (1:100) at 4°C overnight. The slides were washed and incubated with horseradish peroxidase (HRP)-conjugated secondary antibody (1:200) for 1 h at room temperature. The slides were then washed, developed with DAB and counter-stained with hematoxylin. The slides were visualized and captured at ×200 magnification (BX51; Olympus).

### Preparation of tissue homogenates

The heart samples were homogenized in ice-cold RIPA buffer containing protease inhibitor cocktail and phosphatase inhibitor cocktail (both from Roche). The homogenate was then centrifuged at 12,000 x g for 15 min at 4°C. The protein concentration in the supernatant was determined using the Enhanced BCA Protein assay kit (from the Beyotime Institute of Biotechnology, Jiangsu, China). The supernatant was then stored in aliquots at −80°C for further analysis.

### Measurements of myocardial tumor necrosis factor-α (TNF-α) and interleukin-6 (IL-6)

The TNF-α and IL-6 content in the supernatant was measured using quantikine enzyme-linked immunosorbent assay (ELISA) kits (R&D Systems, Minneapolis, MN, USA) following the manufacturer’s instructions. The heart supernatant was added to the respective wells in duplicate followed by incubation with the detection antibody supplemented with substrate and stop solution. The optical density (OD) of the colored product was determined using a spectrophotometer at 450 nm (Microplate Reader, Model 3550; Bio-Rad Laboratories, Hercules, CA, USA). Cytokine amounts were normalized to the protein content and expressed as picograms/milligram protein (pg/mg).

### Western blot anlalysis

Equal amounts of protein (40–60 *µ*g) were separated on 10% sodium dodecyl sulfate-polyacrylamide gel electrophoresis (SDS-PAGE) and transferred onto polyvinylidene difluoride membranes (PVDF, 0.45 *µ*m; Millipore, Billerica, MA, USA). The membranes were blocked in 5% non-fat milk or BSA in 0.05% Tween-20/Tris-buffered saline (TBST) for 1 h at room temperature and incubated with primary antibodies specific for O-GlcNAc (1:800), OGT (1:1,000), OGA (1:400), calcium/calmodulin dependent protein kinase II (CaMKII) (1:800), phosphorylated (p-)CaMKII (1:800), ERK1/2 (1:1,000), p-ERK1/2 (1:1,000), p38 MAPK (1:1,000), p-p38 MAPK (1:1,000) and glyceraldehyde 3-phosphate dehydrogenase (GAPDH) (1:3,000) at 4°C overnight. After washing, the membranes were incubated with HRP-conjugated secondary antibodies (1:4,000) for 1 h at room temperature. The bands were visualized using an enhanced chemiluminescence (ECL) detection kit (Advansta Corp., Menlo Park, CA, USA) and quantified by AlphaEaseFC software (Alpha Innotech, San Leandro, CA, USA).

### Reagents and antibodies

The antibodies for O-GlcNAc (CTD110.6; #sc-59623; mouse monoclonal antibody), NF-κB p65 (sc-109; rabbit polyclonal antibody), CaMKII (M-176; #sc-9035; rabbit polyclonal antibody) and p-CaMKII (22B1; #sc-32289; mouse monoclonal antibody), as well as secondary anti-mouse and anti-rabbit IgG were obtained from Santa Cruz Biotechnology, Inc. (Santa Cruz, CA, USA). Antibodies against OGA (#14711-1-AP; rabbit polyclonal antibody) and OGT (#11576-2-AP; rabbit polyclonal antibody) were from Proteintech (Proteintech Group Inc., Chicago, IL, USA). Antibodies for p38 MAPK (#9212, rabbit polyclonal antibody), p-p38 MAPK (#4511, rabbit monoclonal antibody), ERK1/2 (#4695; rabbit monoclonal antibody), p-ERK1/2 (Thr202/Tyr204) (#4376; rabbit monoclonal antibody), caspase-3 (#9662; rabbit polyclonal antibody), and GAPDH (#5174; rabbit monoclonal antibody) were from Cell Signaling Technology (CST, Beverly, MA, USA). Most high quality reagents were from Sigma-Aldrich (St. Louis, MO, USA).

### Statistical analysis

Statistical analyses were performed using PASW 18.0 software (SPSS Inc., Chicago, IL, USA). Data are presented as the means ± standard deviation (SD). Statistical analysis was performed by one-way analysis of variance (ANOVA), followed by post hoc comparisons [least significant difference (LSD)] or by a Student’s t-test where appropriate. A value of P<0.05 was considered to indicate a statistically significant difference.

## Results

### Changes in arterial blood gas levels, blood pressure and HR

**Figure 1:**
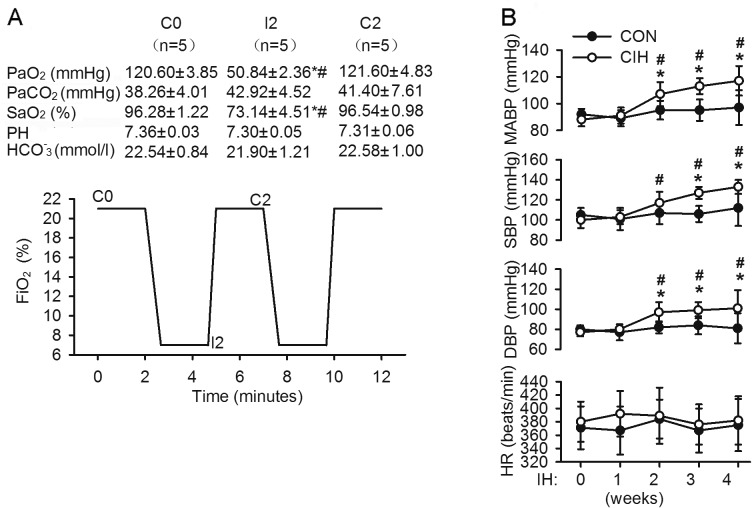
Experimental protocols and changes in systolic blood pressure (SBP), diastolic blood pressure (DBP), mean arterial blood pressure (MABP) and heart rate (HR). (A) Each cycle of intermittent hypoxia (IH) consisted of a 2-min episode of hypoxia (FiO_2_ 6–8%) and a 2-min period of reoxygenation (FiO_2_ 21%). Arterial blood gases were measured at baseline (C0), during the nadir hypoxic portion of the cycle (I2) and at the peak of the air flush (C2). ^*^P<0.05 vs. C0; ^#^P<0.05 vs. C2. (B) Changes in MABP, SBP, DBP and HR in the rats exposed to normoxia or IH. Data are the means ± SD. ^*^P<0.05 vs. corresponding values of the control group (normoxia); ^#^P<0.05 vs. corresponding baseline values.

Over the course of exposure to IH consisting of 2 min of hypoxia followed by 2 min of re-oxygenation, arterial blood gases withdrawn during the most hypoxic portion of the cycle showed a nadir hypoxia within the range of severe OSA, which recovered to the baseline PaO_2_ during the air flush (Fig. 1A). Baseline blood pressure parameters [mean arterial blood pressure (MABP), systolic blood pressure (SBP), diastolic blood pressure (DBP)] and HR were similar between the 2 groups. Exposure to IH for 2 weeks evoked a significant increase in MABP, SBP and DBP, which remained elevated until the end of the 4-week duration of exposure to IH. However, exposure to normoxia failed to cause any significant changes in blood pressure parameters with time. However, neither exposure to normoxia nor IH markedly altered the rats HR during the same observation period (Fig. 1B).

### Changes in cardiac architecture

**Figure 2:**
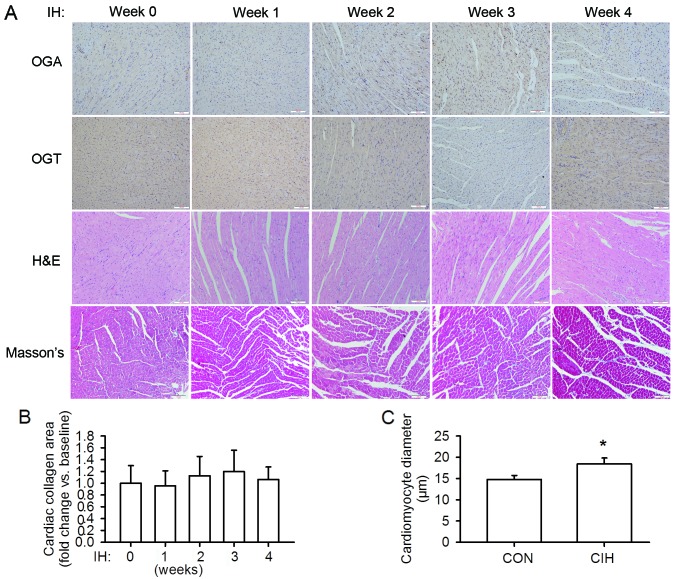
Changes in cardiac architecture and protein expression during the 4 weeks of exposure to intermittent hypoxia (IH). (A) Histopathological analysis and expression of O-GlcNAcase (OGA) and O-GlcNAc transferase (OGT) in the left ventricular tissues. Gradually abnormal myocardial architecture occurred due to exposure to IH, as evidenced by cardiomyocyte disarray and structural disorganization, without significant changes in collagen deposition. OGA and OGT levels changed dynamically during the 4 weeks of exposure to IH. (B) The area of cardiac interstitial collagen prior to exposure to IH was regarded as the baseline value. (C) After 4 weeks of exposure to IH (CIH) there was a significant increase in cardiomyocyte size compared with exposure to normoxia (CON). Scale bar, 100 *µ*m. ^*^P<0.05 vs. normoxia group.

The rats in the IH group gained less body weight than the rats in the normoxia group during the 4-week period of exposure. The ratio of HW/BW and LVW/BW and the indexes of systemic hypertension-induced hypertrophy were significantly higher in the rats in the IH group. The LV tissue sections of the rats in the IH group stained with H&E showed a gradually abnormal myocardial architecture, as evidenced by an increase in cardiomyocyte diameter, cardiomyocyte disarray and structural disorganization. No signigicant changes in the interstitial collagen deposition were observed (Fig. 2 and Table I).

### Changes in LV systolic and diastolic function

Of note, compared to the rats in the normoxia group, the rats exposed to 2 or 3 weeks of IH presented with an augmented LVESP, +dP/dt and −dP/dt, indicative of improved myocardial performance as an outcome of exposure to IH, which then declined from week 4. LVEDP was gradually increased by exposure to IH, with a significant enhancement at week 4. There were no significant differences observed in any of these indexes over time for the rats in the normoxia group (Table I).

### Cardiomyocyte apoptosis and dynamic changes in protein levels of O-GlcNAc, OGA, OGT, ERK1/2 and p38 MAPK

**Figure 3:**
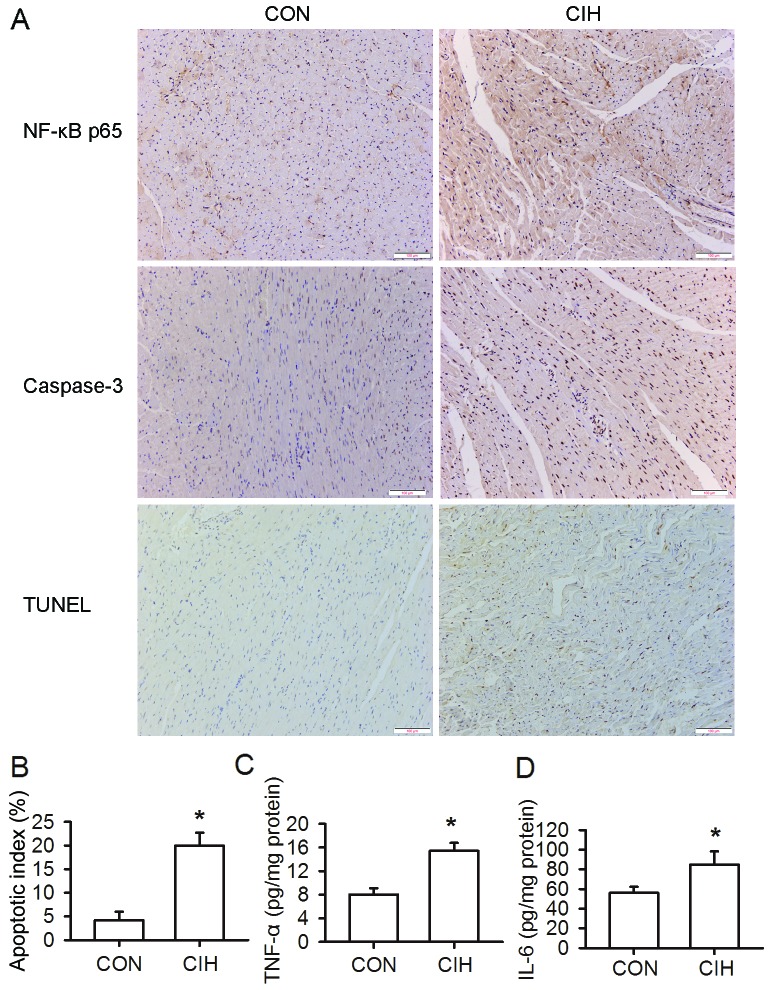
A 4-week exposure to intermittent hypoxia (CIH) led to cardiomyocyte apoptosis and cardiac inflammation. (A) Heart immunohistochemistry and apoptosis assays. (B) CIH induced cardiomyocyte apoptosis. (C and D) CIH increased tumor necrosis factor-α (TNF-α) and interleukin-6 (IL-6) levels. CON, group exposed to 4 weeks of normoxia. Scale bar, 100 *µ*m. ^*^P<0.05 vs. normoxia group.

**Figure 4:**
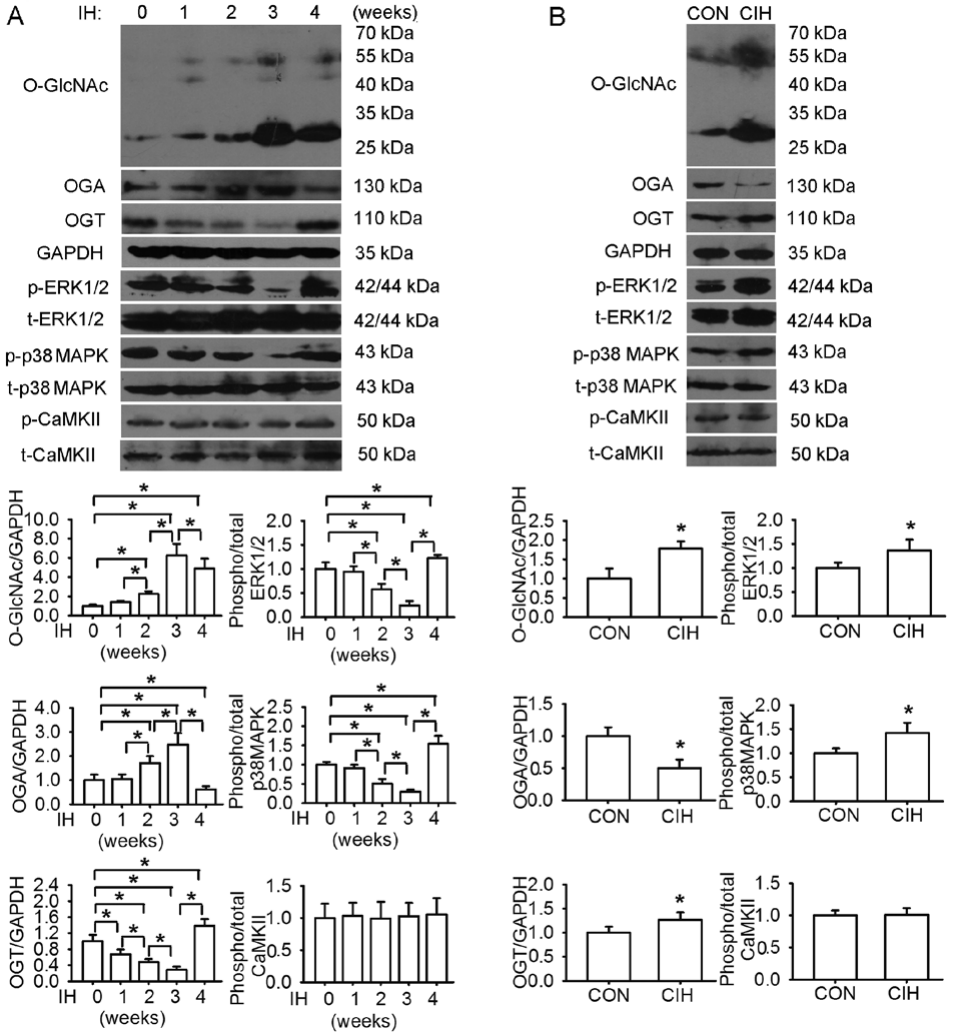
Changes in the protein levels of O-linked β-N-acetylglucosamine (O-GlcNAc), O-GlcNAcase (OGA), O-GlcNAc transferase (OGT), extracellular signal-regulated kinase 1/2 (ERK1/2) and p38 mitogen-activated protein kinase (p38 MAPK) over the course of 4 weeks of exposure to intermittent hypoxia (IH). (A) Protein O-GlcNAc and OGA levels steadily increased following exposure to IH, reaching peak levels at week 3. The expression levels of OGT and the phosphorylation levels of ERK1/2 and p38 MAPK changed in an opposite manner. The levels of phosphorylated CaMKII remained almost unaltered. (B) Compared with exposure to normoxia (CON), 4 weeks of exposure to IH (CIH) augmented O-GlcNAc protein, OGT, phosphorylated ERK1/2 and p38 MAPK levels, accompanied by a decrease OGA in levels. Data are the means ± SD. ^*^P<0.05 between respective groups.

The protein levels of O-GlcNAc in the LV tissues steadily increased following exposure to IH, reaching peak levels at week 3. There was a similar change observed in the OGA levels. The OGT, ERK1/2 and p38 MAPK phosphorylation levels were affected in an opposite manner. The levels of phosphorylated CaMKII remained almost unaltered during the same observation period. In parallel, compared with exposure to normoxia, 4 weeks of exposure to IH augmented the O-GlcNAc protein, OGT, phosphorylated ERK1/2 and p38 MAPK levels, accompanied by a decrease in OGA levels and an increase in cardiomyocyte apoptosis, as well as an increase in the levels of caspase-3, NF-κB p65, TNF-α and IL-6 in the LV tissues (Figs. 3 and 4).

## Discussion

In the present study, we found that exposure to IH induced a significant increase in rat blood pressure from weeks 2 to 4 of exposure, associated with a gradually abnormal myocardial architecture. Compared to the rats in the normoxia group, the rats exposed to IH for 2 or 3 weeks demonstrated an improved LV systolic and diastolic function, which then declined from week 4. Consistently, the protein levels of O-GlcNAc and OGA in the LV tissues steadily increased following exposure to IH, reaching peak levels at week 3. However, the OGT levels and the phosphorylation levels of ERK1/2 and p38 MAPK were affected in an opposite manner. At week 4, the CIH-induced increase in the protein levels of O-GlcNAc and the phosphorylation levels of MAPKs was accompanied by cardiac inflammation, as evidenced by increased cardiomyocyte apoptosis and the cardiac content of NF-κB, inflammatory cytokines and caspase-3. Thus, these findings indicate the possible involvement of protein O-GlcNAc and MAPK signaling in mediating alterations in LV function and cardiac damage during IH.

During the course of the 4 week of exposure to IH, the LV systolic and diastolic capability first increased and then decreased, consistent with previous reports that short-term exposure to IH may exert cardioprotective effects, whereas prolonged exposure to IH appears to exert deleterious effects (24–26). The IH-induced cardiac hypertrophy may exist at first in a compensatory state to augment LV contractility and maintain cardiac output, and then progress to a decompensated state, ultimately evolving to dilated cardiac hypertrophy with LV dysfunction and remodeling (16,27,28). Our findings are in agreement with previous results, indicating that IH protects the isolated heart from reperfusion injury (29) and induces adaptive responses to improve cardiac function during the progression of heart failure (26). Naghshin *et al* (25,26) demonstrated that 4 weeks of exposure to IH induced beneficial cardiac adaptive responses to hypoxia in mice. However, other studies have demonstrated a decline in cardiac function in rats exposed to IH for 4 weeks or longer (16,17,24,28,30), which is consistent with our findings. Thus, there may be a very narrow window of around 3 to 4 weeks of exposure to IH in rodent models during which a transition from physiological adaptations to pathological cardiovascular outcomes occurs.

Intriguingly, in this study, alterations in LV function coincided with the dynamic changes in the protein levels of O-GlcNAc, suggesting a potential mediating role of O-GlcNAcylation. Paradoxically, the expression level of OGT, which catalyzes O-GlcNAc formation, was affected in an opposite manner. However, the expression of OGA, which catalyzes O-GlcNAc removal, was altered in a manner similar to that of O-GlcNAc. In addition to the notion that O-GlcNAc, OGA and OGT levels change with age, other contributing factors, such as changes in enzymatic activity and UDP-GlcNAc concentrations should also be taken into consideration (31). Further studies are warranted to clarify the potential role of these factors in the regulation of cardiac O-GlcNAc levels under IH conditions. Previous studies have demonstrated that an acute increase in O-GlcNAc levels may exert protective effects on the cardiovascular system, whereas a persistent increase may exert adverse effects on the chronic disease status (1,32). In accordance with this, in our study, the protein levels of O-GlcNAc increased rapidly during the first 3 weeks of exposure to IH, contributing to the maintenance of LV function in response to an elevated LV afterload as evidenced by an increase in blood pressure. The sustained increase in O-GlcNAc levels induced by further exposure to IH may lead to the pathogenesis of LV dysfunction and cardiac remodeling. The differential effects of an acute versus a sustained increase in O-GlcNAc levels on cardiac function are possibly due to the modifications of different signaling molecules (1).

Although to the best of our knowledge, the association between O-GlcNAcylation and IH has not yet been investigated to date, the contributing role of O-GlcNAc modification in cardiovascular dysfunction is increasingly recognized (1,2,33). Studies have revealed that an elevated O-GlcNAc modification is linked to the adverse effects of diabetes on the heart by disrupting the cardiomyocyte hypertrophic signaling pathway and altering mitochondrial and mechanical function. Reducing O-GlcNAc levels can restore hypertrophic signaling in a rodent model of diabetes (1,32–34). In addition, an increase in O-GlcNAc levels may contribute to the development and progression of pressure-overload or NFAT-associated heart hypertrophy and infarct-induced heart failure. The properties of O-GlcNAcylation to modulate signal transduction involved in redox, inflammatory and apoptotic processes have also been implicated in the dysregulation of the cardiovascular system (1,2,7,32,33). Accordingly, our finding that CIH increased both O-GlcNAc protein levels and the cardiac content of inflammatory cytokines together with an enhanced cardiomyocyte apoptosis suggests an association between O-GlcNAcyaltion and inflammatory signaling under IH conditions.

The ERK1/2 and p38 MAPK signaling cascades participate in the cardiac remodeling process and in the progression of cardiac dysfunction induced by IH. Béguin *et al* (29) demonstrated that acute IH (AIH) enhanced both ERK1/2 and p38 MAPK phosphorylation levels in the rat myocardium. Inhibitors specific to ERK1/2 and p38 MAPK abolished the AIH-induced cardioprotective effects against ischemic insults. Cardiac hypertrophy in rats subjected to CIH may be attributed to increased p38 MAPK activation (16). The phosphorylation of MAPKs may in turn activate NF-κB, inducing the overproduction of inflammatory cytokines, consequently triggering inflammatory responses, the apoptotic program and the expression of hypertrophy-related genes (16,19). In this study, we found that cardiac remodeling occurred following exposure to IH, which was characterized by cardiac hypertrophy, cardiomyocyte disarray, structural disorganization and increased cardiomyocyte diameter and apoptosis. During the course of exposure to IH, the phosphorylation levels of ERK1/2 and p38 MAPK exhibited dynamic changes, which were almost opposite to those observed in LV function and protein O-GlcNAc levels. Moreover, the increase in the phosphorylation levels of p38 MAPK and ERK1/2 by CIH coincided with LV dysfunction and enhanced cell apoptosis and the increased protein levels of NF-κB and inflammatory cytokines. Taken together, these results indicate that the ERK1/2 and p38 MAPK pathways are possibly involved in the pathological remodeling and impairment of LV function under IH conditions, which may be related to O-GlcNAc modification, since there is an interplay between O-GlcNAcylation and MAPK activation during oxidative stress (1).

CaMKII, the most abundant CaMK in the heart, functions as a nodal signaling molecule in the regulation of cardiac physiology and pathology (35). The activation of CaMKII by phosphorylation has been implicated in cardiac hypertrophy, dilated cardiomyopathy and heart failure (36). However, data on the status of CaMKII activity and the associated changes in CaMKII phosphorylation under IH conditions are controversial, possibly due to the variations in experimental protocols in the aspect of the severity of hypoxia, cycle frequency, exposure duration and cell and tissue types (37–39). Similarly, in this study, we did not observe any significant changes in the phosphorylation level of CaMKII during the course of exposure to IH. CaMKII has 4 isozymes and the antibody used in this study is raised against a synthetic peptide corresponding to residue 286 of the CaMKII α-subunit, whose phosphorylation is known to trigger the autonomous activation of CaMKII. Recently, the oxidation- and O-GlcNAcylation-dependent CaMKII activation has also been found to have critical regulatory implications in cardiac pathologies (40,41). Additional mechanisms for CaMKII activation include the direct modification by nitric oxide (NO) and the interaction with protein partners. Thus, the extent of CaMKII activation in the heart may be partly dependent on phosphorylation (35). It is possible that the protein abundance does not linearly correlate with enzyme activity and the isoform-specific effects may also play a role. Further studies are warranted to determine the activity of CaMKII and to elucidate the potential pathways of CaMKII activation contributing to the regulation of pathological cardiac signaling under IH conditions.

Due to the limitation that the modulation of O-GlcNAc levels *in vivo* in pharmacological or genetical approaches does not result in the desired outcome (33), we failed to investigate the potential regulatory mechanism of O-GlcNAcylation in IH-related cardiac pathophysiology by directly modulating O-GlcNAc levels in our rat model. However, the dynamic changes in the protein levels of O-GlcNAc, OGA and OGT coincided with alterations in LV function, suggesting a possible causal link between O-GlcNAcylation and IH-induced myocardial pathology. Additionally, the extensive interplay between O-GlcNAcylation and phosphorylation, both of which may interfere with redox and inflammatory signaling, points to a need to clarify their regulatory roles in these pathophysiological processes in response to IH. The availability of specific antibodies for O-GlcNAcylated proteins, which can identify the O-GlcNAc-modified sites, would provide new insight into the fundamental mechanisms of O-GlcNAc protein in the modulation of IH-associated cardiac function in future studies. Consequently, O-GlcNAcylation may emerge as a potential therapeutic target in the treatment of OSA-induced cardiac diseases.

In conclusion, the findings from the present study suggested that short-term exposure to IH led to an improvement in cardiac performance; however, a longer period of exposure to IH caused detrimental outcomes. Protein O-GlcNAc levels and cellular signaling involving ERK1/2 and p38 MAPK may act as potential regulators in IH-associated cardiac remodeling and changes in LV function.

## Figures and Tables

**Table I:** Changes in body weight, heart weight and left ventricular function during the 4 weeks of exposure to IH.

		Week 0	Week 1	Week 2	Week 3	Week 4
BW (g)	Sham	211±7 (24)	261±17 (24)^a^	312±23 (24)^a^	327±17 (24)^a^	368±30 (24)^a^
	IH	208±11 (24)	243±11 (24)^a^,^b^	269±18 (24)^a^,^b^	289±57 (24)^a^,^b^	335±51 (24)^a^,^b^
HW/BW	Sham	2.75±0.14 (6)	2.77±0.19 (6)	2.77±0.24 (6)	2.79±0.28 (6)	2.73±0.19 (21)
(×10^3^)	IH	2.73±0.10 (6)	2.81±0.38 (6)	2.93±0.28 (6)	3.00±0.14 (6)^a^	3.08±0.21 (20)^a^,^b^
LV/BW	Sham	2.17±0.12 (6)	2.14±0.20 (6)	2.19±0.19 (6)	2.19±0.22 (6)	2.19±0.14 (21)
(×10^3^)	IH	2.16±0.09 (6)	2.20±0.34 (6)	2.29±0.21 (6)	2.35±0.10 (6)^†^	2.45±0.19 (20)^a^,^b^
LVESP	Sham	108.2±9.7 (6)	111.8±11.0 (6)	114.0±9.5 (6)	119.3±16.1 (6)	118.5±21.9 (13)
(mmHg)	IH	111.0±10.6 (6)	115.6±9.6 (6)	130.5±9.8 (6)^a^,^b^	147.9±11.8 (7)^a^,^b^	139.6±6.9 (8)^a^,^b^
LVEDP	Sham	6.5±3.1 (6)	6.2±2.8 (6)	7.2±3.5 (6)	7.2±3.3 (6)	6.9±4.0 (13)
(mmHg)	IH	6.7±2.6 (6)	7.3±3.0 (6)	7.3±2.6 (6)	8.4±2.5 (7)	14.5±2.0 (8)^a^,^b^
+dP/dt	Sham	6709±960 (6)	6850±1042 (6)	6636±932 (6)	7065±1070 (6)	6993±1037 (13)
(mmHg/sec)	IH	6865±1255 (6)	7089±982 (6)	7739±868 (6)^b^	8255±442 (7)^a^,^b^	5807±729 (8)^a^,^b^
−dP/dt	Sham	7645±864 (6)	7777±1002 (6)	7827±902 (6)	7903±962 (6)	8245±1127 (13)
(mmHg/sec)	IH	7809±867 (6)	8132±1016 (6)	8603±961 (6)	8693±815 (7)	6233±816 (8)^a^,^b^

Sham, normoxia group; IH, intermittent hypoxia; BW, body weight; HW, heart weight; LVW, left ventricle plus septum weight; LVESP, left ventricular end systolic pressure; LVEDP, left ventricular end diastolic pressure; +dP/dt, maximal rate of LV pressure rise in systole; −dP/dt, maximal rate of LV pressure fall in diastole. Values are expressed as the means ± SD. The number of animals examined in each group is indicated in parentheses.

a P<0.05 vs. corresponding baseline values;

b P<0.05 vs. corresponding values of sham group.
