# [Expression of Concern] Alterations in left ventricular function during intermittent hypoxia: Possible involvement of O-GlcNAc protein and MAPK signaling

**DOI:** 10.3892/ijmm.2025.5600

**Published:** 2025-07-30

**Authors:** Xueling Guo, Jin Shang, Yan Deng, Xiao Yuan, Die Zhu, Huiguo Liu

Int J Mol Med 36: 150-158, 2015; DOI: 10.3892/ijmm.2015.2198

Following the publication of this paper, for the histopathological data shown in Fig. 2A, it was drawn to the Editor's attention by a concerned reader that, in the 'OGT' row of data panels, the 'Week 0' and 'Week 1' panels appeared to show an overlapping section of data, such that data which were intended to have shown the results of differently performed experiments appeared to have been derived from the same original source. Moreover, the OGT western blots shown in Fig. 4B were strikingly similar to the blots shown for the p-p38 MAPK experiment in this figure. The authors were contacted by the Editorial Office to offer an explanation for the apparent anomaly in the presentation of the data in this paper, although up to this time, no response from them has been forthcoming. Owing to the fact that the Editorial Office has been made aware of potential issues surrounding the scientific integrity of this paper, we are issuing an Expression of Concern to notify readers of this potential problem while the Editorial Office continues to investigate this matter further.

